# Epidemiology of childhood trauma and its association with insomnia and psychotic-like experiences in Chinese Zhuang adolescents

**DOI:** 10.3389/fpsyt.2022.974674

**Published:** 2022-08-22

**Authors:** Qiaoyue Wei, Yuli Pan, Shengjie Zhang, Wenwen Yin, Qinghong Lin, Shuibo Pan, Chenyangzi Dai, Linhua Zhou, Junduan Wu

**Affiliations:** ^1^Department of Psychology, School of Public Health, Guangxi Medical University, Nanning, China; ^2^Department of Guangxi Zhuang Autonomous Region Center for Disease Control and Prevention, Nanning, China; ^3^Department of Graduate Management, Guangxi Medical University Cancer Hospital, Nanning, China; ^4^Department of Psychology, School of Medicine, Guangxi Medical College, Nanning, China

**Keywords:** childhood trauma, insomnia, psychotic-like experiences, epidemiology, association, adolescents, ethnic minorities

## Abstract

**Background:**

Adolescents who have experienced childhood trauma are more likely to have insomnia and psychotic-like experiences (PLEs) than adolescents from other ethnic groups. However, little is known about the youth of ethnic minorities. This study aimed to investigate the epidemiology of childhood trauma and its relationship with insomnia and PLEs in Chinese Zhuang adolescents, focusing on the role of a specific type of trauma and accumulation.

**Methods:**

A questionnaire of Childhood Trauma Questionnaire-Short Form (CTQ-SF), Athens Insomnia Scale (AIS), and Chinese Version Community assessment psychic experiences-8 (CCAPE- 8) were all completed by 1,493 Chinese Zhuang adolescents. Chi-square and multivariate logistic regression analyses examined the association between childhood trauma and insomnia/PLEs.

**Results:**

The incidences of emotional abuse (EA), physical abuse (PA), sexual abuse (SA), emotional neglect (EN), and physical neglect (PN) occurred at rates of 5.63, 5.02, 6.56, 23.98, and 33.15%, respectively. EA, SA, EN, and PN were all positively related to insomnia (OR: 1.314–7.720, all *p* < 0.05). EA and SA were positively associated with PLEs (OR: 2.131–3.202, all *p* < 0.001). Adolescents who had experienced three or more types of traumas were more likely to have insomnia (OR = 6.961, *p* < 0.001) and PLEs (OR = 3.558, *p* < 0.001).

**Conclusion:**

The most common type of childhood trauma is PN. Childhood trauma has the primary effect on insomnia/PLE. A significant dose-response relationship was found between Childhood trauma and insomnia/ PLEs. This association varied depending on the type and accumulation of exposure.

## Introduction

Childhood trauma refers to various types of neglect and abuse that a person may experience as a child (1), including emotional abuse (EA), physical abuse (PA), sexual abuse (SA), emotional neglect (EN), and physical neglect (PN) (2). According to the World Health Organization (WHO) (3), more than one-third of the population has experienced childhood trauma, with a much higher prevalence among ethnic minority children (4). Further studies suggest that childhood trauma has a significant negative impact on a child's physical and psychological health throughout adolescence and even the entire life course (5) and may be responsible for mental disorders (3). It is worth noting that a single type of trauma can be misleading (6), as clients experience only this type of trauma in their lifetime, which may be a rare condition. However, the classification of trauma is an essential tool for a good assessment, as different types of trauma produce different symptoms. Researchers found a dose-response relationship (7, 8), between physical and mental conditions, with those exposed to a higher number of traumas having a greater risk of physical and mental illness. Thus, in this article, we explored not only the contribution of individual trauma types to determine whether specific trauma types were driving the association between trauma and health outcomes but also the cumulative effect of childhood trauma on health outcomes to evaluate the dose-response relationship.

According to a recent WHO report, 23, 36, and 16 % worldwide have experienced PA, EA, or PN (9). Previous research on childhood trauma has mostly focused on adults, with only a few studies focusing on adolescents. Adolescence is a critical period of behavioral, cognitive, mental, and physical development and a period of potential vulnerability (10). Furthermore, one of the especially neglected aspects of trauma assessment is ethnicity (6). Significant disparities in childhood neglect and abuse have been observed due to racial and ethnic differences (11, 12). Zhuang, is the largest one of Chinese minorities, with the majority growing up in a remote village far from the school grounds. Besides, they may face incorrect parenting styles due to their unique culture, spoken language, and lower parental education (13, 14). As a result, they have much higher rates of physical, mental (15, 16) and neglect than those in Han areas (17). However, few existing studies focused on ethnic minority adolescents, and it remains unclear if the prevalence of childhood trauma is higher or different due to ethnic minorities. The current study aims to fill this gap.

Insomnia is common in adolescents; it is one of the top health concerns for parents (18) and global public health concerns (19). Researchers found that insomnia is associated with increased mental and physical health consequences in adolescents, including depression (20), anxiety (21), obesity (22), alcohol abuse (23), and suicidal behavior (24). The causes of insomnia are complex (25). Current evidence indicates that adolescents who have experienced childhood trauma are more likely to experience insomnia (26), with a clear dose-response relationship throughout the life cycle (27). Although these studies suggest a link between childhood trauma and insomnia, there is a lack of research on the potential associations between each specific type of trauma and insomnia in adolescents.

Psychotic-like experiences (PLEs) resemble the positive symptoms of psychosis and are relatively common in adolescents (28). While PLEs have been considered as the mildest manifestation of psychosis tendencies, evidence exists that they are also associated with a wide range of mental health problems (29). For example, PLEs, including unusual beliefs, perceptual abnormalities, and persecutory ideation during childhood, are positively associated with psychotic disorders, depression, and anxiety in adulthood (30). A number of studies have found that childhood trauma is associated with an increased risk of psychotic disorders (31). As suggested by previous research, specific types of traumas, such as witnessing violence (32) or emotional abuse (33), may be strongly associated with PLEs.

Currently, the co-occurrence of insomnia and PLEs has been strongly supported in a large number of articles, including community and clinical populations (34, 35). Furthermore, insomnia is a common prodrome feature of psychosis or PLEs (36). On the other hand, specific sleep structures associated with insomnia, may be hallmarks of PLEs, such as reduced slow-wave sleep (37). Moreover, neurotransmitter changes in psychotic symptoms may also induce insomnia through excessive dopamine activity (38). Although the relationship between insomnia and PLEs has been explained no matter epidemiological investigation or physiological mechanism research, some environmental risk factors, such as childhood trauma, still need to be identified simultaneously. Further, evaluating such symptoms in adolescents is essential as adolescence is a particularly crucial period for both insomnia and PLEs (29).

In summary, we designed this study to investigate: (1) the prevalence of specific types of childhood trauma in Zhuang adolescents; and (2) the relationship between multi-types of trauma and insomnia and PLEs. We also hypothesized a dose-response relationship between childhood trauma with insomnia and PLEs.

## Methods

### Participants

A cross-sectional study was conducted in September 2019 at three schools (two junior highs and a senior high school) in Guangxi, China. We selected freshman students to reduce the recall bias of traumatic childhood events. The inclusion criteria were the age of 12 to 18 years and must be of Zhuang nationality. Exclusion criteria were a history of psychological disease, and the use of psychoactive drugs. Finally, 1,493 questionnaires were included after excluding those who refused to participate in the study or failed to submit their completed questionnaires. Among them, 712 (47.7%) were boys, while 781 (52.3%) were girls. The average age of participants was 14.9 ± 1.5 years old. The studies involving human participants were reviewed and approved by the Institutional Ethical Committee of Guangxi Medical University (approval number: 20160302-13). The participants and their parents or legal guardians provided written informed consent to participate in this study.

### Measurements

#### Childhood trauma questionnaire-short form

The CTQ-SF created by Bernstein is a 28-item retrospective self-reporting screening measure for a history of neglect and abuse in childhood, with five categories for children 12 years and older (39). The five subscales included were emotional abuse (EA), physical abuse (PA), sexual abuse (SA), emotional neglect (EN), and physical neglect (PN). Each subscale contains five items rated on a 5-point Likert scale (1 = “never true” to 5 = “very often true”). To estimate the incidence of each type of childhood trauma, we used the cut-off values EA ≥ 13, PA ≥ 10, SA ≥ 8, EN ≥ 15, or PN ≥ 10, suggested by Bernstein (40). These cut-off scores showed a good validity in Zhuang people (41, 42) and Chinese adolescents (43, 44). The current sample for the CTQ-SF was calculated using Cronbach's alpha with a value of α = 0.816. In this article, the summing of trauma exposure referred to the sum of five indicators of childhood trauma: EA, PA, SA, EN, and PN.

#### Chinese version community assessment psychic experiences-8

The 42-item CAPE scale was proposed by Stefanis et al. (45) which was used to assess the positive, negative and depressive dimensions of psychotic symptoms in the past 12 months. Wang et al. (46) chose eight common items from Arseneault et al. (47) study to investigate the occurrence frequency of adolescents' PLEs, owing to a large number of positive items in the questionnaire and the lack of representativeness in the adolescent population. The simplified CAPE-8 has better reliability and validity with the Cronbach α = 0.768. Participants were asked if they had ever experienced each PLE (e.g., “*Have you ever seen something that was not there that other people could not see?”; “Have you ever heard any voices that other people said did not exist?*” etc.). Each frequency question is answered on the 4-point Likert scale (0 = “never” to 3 = “almost always”), and any items that reach the top of the scale are considered positive. The present sample for the CCAPE- 8 was calculated using Cronbach's alpha with a value of α = 0.715.

#### Athens insomnia scale

The AIS is a self-report questionnaire consisting of 8 items designed to assess participants' insomnia symptoms. Each item is scored on a 4-point Likert scale (0 = “no problem” to 3 = “very severe problem”). The total score ranges from 0 to 24, and the cut-off score for insomnia is 6 (48). According to reports, AIS has good reliability and validity in Chinese adolescent groups (49, 50). The current sample for the AIS was calculated using Cronbach's alpha with a value of α = 0.790.

### Statistical analysis

Statistical analyses were performed using the SPSS Statistics, version 23 (IBM, Armonk, NY, USA). The significant level was set at *p* < 0.05. Before proceeding with the analysis, statistical assumptions were performed on each variable, including demographic and psychological characteristics. All assumptions were met for the chi-square and multivariate logistic regression tests.

Chi-square analyses were used to test demographic differences in different types of childhood trauma. Multivariate logistic regressions analyses examined the association between childhood trauma and insomnia/PLEs. The strength of the association was calculated by odds ratio (OR) and 95% confidence interval (CI).

Given the small sample size, a priori analysis was performed using G^*^ Power software (version 3.1) (51) with a target power of β = 0.80 and an alpha level of 0.05 to determine a large effect size (0.15) (52). A *post hoc* power analysis was also performed, which indicated that statistical power (power = 0.92) was adequate.

## Results

### The prevalence and demographics of childhood trauma

The final analyses included 1,493 participants. The incidences of EA, PA, SA, EN, PN, 1-2 types of traumas, and ≥3 types of traumas were 5.63, 5.02, 6.56, 23.98, 33.15, 41.26, and 6.22%, respectively (Table 1). The proportion of boys was slightly higher than girls (52.31 vs. 47.67%). Their age ranged from 12 to 16 years. Table 2 presents the comparison of different types of childhood trauma in demographics.

**Table 1:** The proportion of different types / numbers of childhood trauma (*n* = 1,493).

**Variables**	***n* (%)**
EA	84 (5.63)
PA	75 (5.02)
SA	98 (6.56)
EN	358 (23.98)
PN	495 (33.15)
1–2 types of traumas	616 (41.26)
≥3 types of traumas	93 (6.22)

**Table 2:** Comparison of different types of childhood trauma in demographics.

	**EA**	**PA**	**SA**	**EN**	**PN**
**Gender**					
Boys (*n* = 712)	27 (3.79)	39 (5.48)	54 (7.58)	154 (21.63)	221 (31.04)
Girls (*n* = 781)	57 (7.30)	36 (4.61)	44 (5.63)	204 (26.12)	274 (35.08)
*χ^2^*	8.623**	0.588	2.310	4.121*	2.748
**Marital status of parents**					
Stable (*n* = 1,312)	69 (5.26)	61 (4.65)	82 (6.25)	289 (22.03)	460 (35.06)
Unstable (*n* = 181)	15 (8.29)	14 (7.73)	16 (8.84)	69 (38.12)	85 (46.96)
*χ^2^*	2.747	3.174	1.739	22.601***	9.718***
**Only child**					
Yes (*n* = 319)	18 (5.64)	19 (5.96)	20 (6.27)	66 (20.69)	103 (32.29)
No (*n* = 1,174)	66 (5.62)	56 (4.77)	78 (6.64)	292 (24.87)	442 (37.65)
*χ^2^*	0.000205	0.740	0.057	2.407	3.110
**Place of residence**					
Rural (*n* = 1,121)	61 (5.44)	50 (4.46)	72 (6.42)	281 (25.07)	440 (39.25)
City (*n* = 372)	23 (6.18)	25 (6.72)	26 (6.99)	77 (20.70)	105 (28.23)
*χ^2^*	0.289	2.990	0.146	2.923	14.647***
**Parental absence**					
Yes (*n* = 844)	60 (7.11)	46 (5.45)	62 (7.34)	206 (24.41)	305 (36.14)
No (*n* = 649)	24 (3.70)	29 (4.47)	36 (5.55)	152 (23.42)	240 (36.98)
χ^2^	8.039**	0.741	1.936	0.196	0.112
**Exercise situation**					
Yes (*n* = 1,219)	62 (5.09)	58 (4.76)	78 (6.40)	263 (21.57)	418 (34.29)
No (*n* = 274)	22 (8.03)	17 (6.2)	20 (7.30)	95 (34.67)	127 (46.35)
*χ^2^*	3.649	0.981	0.296	21.050***	14.039***
**Smoking**					
Yes (*n* = 136)	14 (10.29)	13 (9.56)	16 (11.76)	48 (35.29)	59 (43.38)
No (*n* = 1,357)	70 (5.16)	62 (4.57)	82 (6.04)	310 (22.84)	486 (35.81)
χ^2^	6.140*	6.451*	6.599*	10.510**	3.055
**Drinking**					
Yes (*n* = 215)	18 (8.37)	16 (7.44)	26 (12.09)	57 (26.51)	74 (34.41)
No (*n* = 1,278)	66 (5.16)	59 (4.62)	72 (5.63)	301 (23.55)	471 (36.85)
χ^2^	3.567	3.079	12.520***	0.884	0.471

***p < 0.001, **p < 0.01, *p < 0.05.

### Multivariate logistic regression analysis of insomnia/ PLEs

#### Associations between childhood trauma and insomnia

In addition to controlling for demographic factors, such as gender, parental marital status, having an only child, place of residence, parental absences, smoking, and drinking, we also controlled for exposure to other types of trauma (for example, the relation of PA on insomnia while accounting for EN, EA, SA and PN), as the high co-occurrence of different traumas makes it difficult to tell whether these effects are attributable to the specific type of trauma. Multivariate logistic regression analysis showed that gender, smoking, EN, EA, SA, and PN were all positively related to insomnia (OR: 1.314–7.720, all *p* < 0.05). Those who experienced three or more types of traumas (OR = 6.961, *p* < 0.001) or 1–2 types of traumas (OR = 1.690, *p* < 0.001) had a significantly higher risk of insomnia than those who had no traumas (Tables 3, 4).

**Table 3:** Multivariate logistic regression of different types of childhood trauma on insomnia / PLEs [OR (95% CI)]^a^.

**Variables**	**Insomnia**	**PLEs**
EA (ref. = none)	7.720 (3.217–18.530)***	3.202 (1.915–5.354)***
PA (ref. = none)	0.853 (0.472–1.543)	1.498 (0.874–2.568)
SA (ref. = none)	1.623 (1.006–2.619)*	2.131 (1.373–3.307)***
EN (ref. = none)	1.547 (1.157–2.068)**	0.913 (0.666–1.250)
PN (ref. = none)	1.314 (1.027–1.681)*	0.981 (0.748–1.287)
Gender (ref. = male)	1.538 (1.227–1.928)*	1.080 (0.845–1.381)
Marital status of parents (ref. = stable)	1.390 (0.982–1.969)	1.207 (0.846–1.724)
Only child (ref. = no)	1.201 (0.911–1.582)	1.033 (0.768–1.389)
Place of residence (ref. = rural)	1.090 (0.845–1.407)	1.688 (1.293–2.203)***
Parental absence (ref. = no)	1.083 (0.871–1.346)	1.222 (0.961–1.553)
Exercise situation (ref. = yes)	1.281 (0.965–1.701)	1.342 (0.997–1.805)
Smoking (ref. = no)	2.565 (1.621–4.058)***	1.139 (0.728–1.780)
Drinking (ref. = no)	1.239 (0.870–1.765)	0.979 (0.667–1.410)

^a^The reference category for the dependant variables were none (without insomnia). ***p < 0.001, **p < 0.01, *p < 0.05. OR, odds ratio; CI, confidence interval; Ref, Reference.

**Table 4:** Multivariate logistic regression of the number of types of childhood trauma on insomnia/ PLEs [OR (95% CI)]^a^.

**Variables**	**Insomnia**	**PLEs**
Types of trauma (ref. = no)		
1–2 types of traumas	1.690 (1.356–2.107)***	1.070 (0.837–1.368)
≥3 types of traumas	6.961 (3.702–13.087)***	3.558 (2.267–5.586)***
Gender (ref. = male)	1.596 (1.276–1.998)***	1.079 (0.847–1.375)
Marital status of parents (ref. = stable)	1.395 (0.987–1.972)	1.179 (0.829–1.677)
Only child (ref. = no)	1.220 (0.927–1.607)	1.064 (0.794–1.427)
Place of residence (ref. = rural)	1.090 (0.846–1.404)	1.698 (1.304–2.211)***
Parental absence (ref. = no)	1.093 (0.881–1.358)	1.240 (0.977–1.574)
Exercise situation (ref. = yes)	1.297 (0.978–1.719)	1.307 (0.974–1.754)
Smoking (ref. = no)	2.553 (1.614–4.037)***	1.134 (0.728–1.764)
Drinking (ref. = no)	1.260 (0.886–1.792)	1.023 (0.708–1.477)

^a^The reference category for the dependant variables were none (without insomnia).

***p < 0.001. OR, odds ratio; CI, confidence interval; Ref, Reference.

#### Associations between childhood trauma and PLEs

In addition to controlling for demographic factors, we also controlled for exposure to other types of trauma (for example, the relation of PA to insomnia while accounting for EN, EA, SA, and PN). Multivariate logistic regression analysis revealed that place of residence, EA, and SA were all positively related with PLEs (OR: 2.131–3.202, all *p* < 0.001). Those who reported three or more types of traumas (OR = 3.558, *p* < 0.001) were significantly more likely to develop PLEs than those who did not report any traumas (Tables 3, 4).

In Summary, EA, SA, and having experienced three or more types of traumas are all predominant correlated with insomnia and PLEs.

## Discussion

### The prevalence and demographics of childhood trauma

There are several main findings on the prevalence of childhood trauma in the present study. Firstly, we found that the highest rate of trauma was PN, followed by EN, SA, EA, and PA. This ranking order was consistent with the previous research reported in China (31, 43) while inconsistent with western research (53). This analysis indicates that many Chinese parents still widely consider physical neglect as a legitimate means of inculcating child discipline (54). Furthermore, girls had significantly higher rates in EN and EA than boys, consistent with previous research (55, 56). In contrast, Xiao et al. (27) found that the prevalence of EN and EA (also assessed by the CTQ-SF) was higher in boys than in girls. However, there is disagreement about the gender characteristics of childhood trauma in the current study. Thus, future studies should be further divided into subgroups to understand the gender characteristic of childhood trauma (57). Thirdly, those who reported that unstable parental relations had a high incidence of EN and PN. One explanation for this finding is that emotional turmoil of a high-conflict marriage frequently overlooks concern for children when parents are constantly fighting (58), resulting in the problem that a child is so neglected by parents. Moreover, we found that adolescents living in rural areas were more vulnerable to PN than those in cities. Generally, this group's parents likely face lower socioeconomic status, schooling, and mental health education (57). According to the family stress model (FSM) (59), stressors such as socioeconomic strains cause psychological distress (e.g., depression and family dysfunction), which results in non-optimal parenting (e.g., lack of warmth and support). Furthermore, adolescents with parental absence are more likely to suffer from EA. For this group, parents tended to leave their children to go to the cities in search of better jobs because of limited local jobs (60, 61), which means children are separated from their parents, which can be emotionally damaging (62). And children who grow up without parental involvement, protection, or emotional support are more likely to experience emotional abuse (63). Those who reported smoking, drinking, and not exercising had a higher rate of various types of childhood trauma, reminding us to pay more attention to the impact of bad living habits on mental health (64).

### Association between the different types of childhood trauma and insomnia/ PLEs

In the current study, we found that all four types of trauma except PA significantly increased the risk of insomnia, indicating that dimensional models are not important, particularly with childhood trauma where co-exposure is highly prevalent (65). On the other hand, this result is consistent with most recent studies, which support that childhood neglect and abuse may confer risk for insomnia (66, 67). Combined analyses of trauma, stress, sleep neurobiology, and psychophysiology offer possible insights (68). Some evidence suggests that exposure to trauma triggers a strong stress/fear response that causes hyperarousal in specific brain regions (e.g., amygdala, prefrontal cortex, reticular activating system) (69). As a result, during sensitive developmental periods, overactivation of the hypothalamic-pituitary-adrenal (HPA) axis persists, preventing it from maintaining normal sleep patterns during stressful times and increasing the risk of insomnia (70). Adolescents who had experienced emotional abuse were nearly eight times more likely to report insomnia than those who had not experienced emotional abuse. This suggests that specific types of trauma were driving the association between trauma and health outcomes (8). We can also speculate that EA is a chronic stressor that perpetuates a pattern of chronic stress in subsequent sleep throughout a child's life, possibly for a decade or more.

Besides, we found that EA and SA were risk factors for PLEs. These findings are consistent with previous research suggesting that childhood trauma (33), particularly those characterized by abuse, can cause negative cognition about oneself and the world, resulting in psychological and/or physical vulnerability and positive psychotic symptoms such as psychotic experiences (71). Neurodevelopmental changes related to the HPA axis may be the underlying mechanism of psychotic symptoms in children who have experienced childhood trauma (72).

Emotional abuse, the most harmful form of childhood trauma, was also the strongest predictor of PLEs (3), which may lead to a decreased cortisol response in the HPA axis and thus induce childhood psychotic symptoms (73). Another explanation was that self-esteem and fearful attachment were mediators between emotional abuse and PLEs (74, 75). Notably, no single mechanism explains the observed relationship between childhood trauma and insomnia. More likely, these mechanisms interact with one another, involving biological and social pathways (76). Furthermore, the emergence of an SA-PLEs co-occurrence class was not unexpected. In this context, several studies have corroborated the association that PLE may be associated with SA in some people. Biological, attachment, dissociative, and cognitive perspectives have been used to explain the relationship between SA and PLEs (77).

### Association between the number of types of childhood trauma and insomnia/ PLEs

Our results indicated that the greater childhood trauma, the higher is his/her risk for insomnia during puberty, with the aOR increasing from 1.690 to 6.961 as the number of cumulative childhood trauma increased from one or two to three or more, respectively. This finding is similar to previous studies, which also found that the risk of insomnia in adolescence increases with additional exposure to a traumatic event (78). Simultaneously, this result confirms the cumulative perspective of trauma, indicating that any preventive measures aimed at detecting and reducing it should target a broad range of childhood trauma rather than a single type (79).

Experiencing one or two traumas did not increase the risk of PLEs, indicating that most people can self-regulate the mental state related to low levels of traumas (80). Furthermore, suffering three or more types of traumas increases the risk of PLEs, which parallels previous research that showed a clear dose-response association between childhood trauma and psychotic experiences (81). However, the single-factor analysis showed that only EA and SA are associated with PLEs. We can speculate the difference between “1-2 types of traumas” and “≥3 types of traumas” just caused by EA or SA, which is an interesting founding. Although it is unclear why there is such interesting results at this stage, it can emphasize the importance of considering separately the different forms of trauma to PLEs (82). Meanwhile, it is necessary to further discuss the dose-response relationship. The mechanism of this association could involve multiple emotions of childhood trauma, the cognitive models of psychosis and biological models of stress (82), including improved emotional response and poor emotion regulation abilities (83), and deficits in cognitive control (84), as well as dysregulation of dopaminergic and glutamatergic systems (85).

We also found EA, SA and suffering three or more types of traumas are all predominant correlated with insomnia and PLEs. According to previous research, the relationship between these variables and insomnia / PLEs may mediated by lack of resilience or negative emotions, such as anxiety, worry and depression (70, 86, 87). Consequently, this suggested that insomnia / PLEs could be prevented if exposure to traumas were removed or interventions were developed to mitigate the risk pathways linking childhood trauma to insomnia / PLEs.

Generalize in summary, the type and number of childhood trauma increase the risk of insomnia and PLEs. Different childhood trauma has different impacts on insomnia and PLEs, showing a dose-response association. This could imply that childhood trauma was distal risk factor that harmed adolescents' physical and mental development. PN has the highest rate of trauma, indicating that physical neglect should be prioritized during the vulnerable age period. Childhood traumas affect insomnia and PLE in adolescents negatively and thus should be considered when screening for adolescents' mental health.

## Limitation

This study has several limitations. Above all, these findings are based on a cross-sectional survey, which cannot reveal its causal association with insomnia/PLEs. Since the assessments of childhood trauma, insomnia, and PLEs were based on the self-reported, participants may also have recall bias, with insomnia not corroborated by actigraphy, polysomnography, or medical records. Such reports may lead to over-/under- reporting. Besides, the dichotomous approach (i.e., exposed versus unexposed) we used to estimate childhood trauma may obscure the impact of its intensity or chronicity. Furthermore, determining the precise timeline of childhood trauma, collecting more information on sociodemographic characteristics and structural environment will make our studies more interesting and aid reproducibility and transparency in describing the sample composition. Finally, our research has not yet revealed enough the ethnic differences due to lack of ethnic comparisons.

## Conclusion

For Chinese Zhuang adolescents, PN is the most common type of childhood trauma. Childhood traumas of various types have different effects on insomnia and PLE. As the number of childhood trauma increases, the risk ratio for insomnia and PLE also increases. Consequently, in order to reduce the burden of insomnia and mental disorders, Chinese parents should be educated about childhood neglect and abuse, and educators and mental health professionals should provide targeted interventions for adolescents who have experienced childhood trauma.

## Data availability statement

The original contributions presented in the study are included in the article/supplementary material, further inquiries can be directed to the corresponding author.

## Ethics statement

The studies involving human participants were reviewed and approved by the Institutional Ethical Committee of Guangxi Medical University (approval number: 20160302-13). The participants and their parents or legal guardians provided written informed consent to participate in this study.

## Author contributions

QW, YP, and SZ designed the study and supervised the data collection. WY, QL, SP, CD, and LZ undertook data collection. QW conducted the statistical analyses and drafted the manuscript. JW participated in coaching as a leader. All authors contributed to and have approved the final manuscript.

## Funding

This work was funded by the National Nature Science Foundation of China (No. 81660569). The funders had no role in study design, data collection and analysis, decision to publish, or preparation of the manuscript.

## Conflict of interest

The authors declare that the research was conducted in the absence of any commercial or financial relationships that could be construed as a potential conflict of interest.

## Publisher's note

All claims expressed in this article are solely those of the authors and do not necessarily represent those of their affiliated organizations, or those of the publisher, the editors and the reviewers. Any product that may be evaluated in this article, or claim that may be made by its manufacturer, is not guaranteed or endorsed by the publisher.
